# Resistance to COVID-19 vaccination and the social contract: evidence from Italy

**DOI:** 10.1038/s41541-023-00660-8

**Published:** 2023-04-22

**Authors:** Sarah E. Kreps, Douglas L. Kriner

**Affiliations:** Department of Government, Cornell University, Ithaca, NY USA

**Keywords:** Public health, Vaccines

## Abstract

Confronted with stalled vaccination efforts against COVID-19, many governments embraced mandates and other measures to incentivize vaccination that excluded the unvaccinated from aspects of social and economic life. Even still, many citizens remained unvaccinated. We advance a social contract framework for understanding who remains unvaccinated and why. We leverage both observational and individual-level survey evidence from Italy to study the relationship between vaccination status and social context, social trust, political partisanship, and adherence to core institutional structures such as the rule of law and collective commitments. We find that attitudes toward the rule of law and collective commitments outside the domain of vaccination are strongly associated with compliance with vaccine mandates and incentives. Partisanship also corresponds with vaccine behaviors, as supporters of parties whose leaders criticized aggressive policies to incentivize or mandate vaccination and emphasized individual liberty are least likely to comply. Our findings suggest appeals emphasizing individual benefits may be more effective than appeals emphasizing collective responsibility.

## Introduction

In May 2020, as scientists across the world raced to develop a vaccine against a novel coronavirus with unprecedented speed, a viewpoint article in the *Journal of the American Medical Association* warned of an equally daunting challenge: “The mere availability of a vaccine is insufficient to guarantee broad immunological protection; the vaccine must also be acceptable to both the health community and general public”^1^.

Researchers across the world answered the implicit call to understand the behavioral side of vaccination, including the sources of vaccine hesitancy. Most of these studies have focused on a range of factors including the role of COVID-specific beliefs and fears^2–4^, such as the perceived severity of side effects and concerns over the use of novel mRNA technologies; as well as trust in the institutions directly responsible for shaping pandemic policy^5–7^; and demographic variation in vaccine hesitancy^8,9^ including, in many contexts, an emphasis on partisan political divides^10–14^.

The highly-contagious Omicron variant presented new public health challenges that, coupled with plateauing vaccination rates, prompted many governments to adopt stronger policy measures, including mandates, to incentivize vaccination^15–17^. While previously studied factors associated with vaccine hesitancy may still be relevant in explaining continued resistance in the face of financial penalties and vaccine requirements to patronize restaurants and ride public transportation among other activities, the expanded role of the state in incentivizing and even mandating vaccination has raised the possibility that a broader set of factors may account for individuals’ decisions to continue holding out on vaccination.

In this research, we investigate compliance with vaccine mandates and incentives as adherence to a social contract in which citizens have a moral obligation to protect vulnerable others^18^. If vaccination decisions are part of a social contract, then social trust, adherence to collective commitments, and the mix of trusted elite messages concerning such commitments that citizens receive, should strongly influence vaccination behaviors. The argument builds on recent research employing laboratory experiments showing that vaccinated individuals treat other vaccinated individuals more generously than unvaccinated subjects who remain outside of the social contract^18,19^. While previous work emphasizes how vaccination status and therefore compliance or non-compliance with the social contract affects subsequent inter-personal behavior, we extend the logic to examine whether variation in individuals’ perceptions of social obligations and willingness to abide by the social contract also influences decisions to vaccinate or not.

We focus on Italy, as it was the initial epicenter of the pandemic in Europe and implemented some of the strictest measures to incentivize vaccination, mandating vaccination for all residents fifty years of age and older and requiring proof of vaccination and receiving a booster to receive the “super” green pass (*rafforzato*), which from December 2021 through April 2022 was required to go to work, eat in restaurants, and participate in almost any facet of social life^20^. Both policies fit Attwell et al’s^21^ definition of a vaccine mandate, which can either require all or a subset of the population to vaccinate or require vaccination to access fundamental services or societal benefits. Past research has shown the efficacy of such measures in spurring vaccination^22,23^. However, at the time our survey was in the field in March 2022 while the green pass was still in force, a significant percentage continued to resist, despite facing among the most stringent measures in the world to mandate or incentivize vaccination. Our study seeks to understand the factors underlying this entrenched hesitancy. Specifically, the analyses below empirically test a series of five hypotheses describing observable implications that would be consistent with a vaccination as social contract perspective.

A social contract logic of vaccine acceptance suggests that both social context and inter-personal trust should be significantly associated with vaccination. An important strand of previous research argues that social context often plays a critical role in shaping individuals’ social behaviors. Since Putnam’s^24^ seminal *Making Democracy Work*, a large literature has explored the linkages between variation in regional social capital – which Putnam defines as social networks and the norms of trust and reciprocity that arise through them – and a range of governmental^25^, economic^26^, and health outcomes^27^. Individuals embedded in communities with high levels of social capital and dense networks of social trust and reciprocity – regardless of their own backgrounds and characteristics – may be more likely to abide by the social contract than those who live in areas poorer in social capital. This leads to our first hypothesis:

H1: Regional social capital will be negatively associated with the percentage of the population that has not received a COVID-19 vaccine.

Beyond social context, a social contract perspective also suggests that individual-level variation in social trust should also correlate with vaccine uptake. Individuals with greater levels of social trust may perceive a greater duty to protect the community and greater faith that others will also comply with government mandates and vaccinate. The former encourages vaccination regardless of the actions of others. The latter encourages vaccination by raising one’s estimates of the likelihood that the collective good will be achieved. Individuals who have higher levels of social trust are more likely to see and expect the collective benefits that societal vaccination affords^28–30^. After all, the collective benefit will only be achieved if enough individuals have high levels of social trust that in turn encourage them to resist the temptation to free-ride and not vaccinate^31,32^. This yields a second hypothesis:

H2: Individuals with higher levels of social trust will be less likely to be unvaccinated against COVID-19.

Individuals enter into the social contract through the state, which also enforces the contract. In the case of vaccination against COVID-19, many governments have implemented a range of policies from strong inducements to outright mandates to encourage compliance. As a result, institutional trust should also be associated with individuals’ willingness to abide by the vaccination social contract.

In countries where vaccine mandates are written in law (as they are in Italy^20^), we would expect to see strong associations between individual attachments to the rule of law and compliance with the vaccination contract. Legal scholars argue that attachments to the rule of law are core commitments that shape and inform other political attitudes and behaviors^33–35^. Those most committed to the rule of law^36^ will be the most likely to comply, and those least committed will be the most likely to remain unvaccinated. This generates a third hypothesis:

H3: Individuals with stronger commitments to the rule of law will be less likely to be unvaccinated against COVID-19.

Whether an individual views compliance with policies that incentivize or mandate vaccination as part of a social contract may be critically reinforced – or sometimes challenged^37^ – by the actions and cues transmitted to citizens by political elites. For many citizens, partisan attachments are a form of social identity that help them make sense of the political world^38^. As such, when trying to understand which policies are in the national interest and which are not, and even when thinking about the expectations and duties of citizenship such as vaccination, many citizens may logically look to the cues transmitted by trusted co-partisan elites^12,39–41^.

Past research has shown evidence of significant partisan divides in vaccination, particularly in the United States^42^ but also in other countries^43^. Most prior research has identified ideological divides, with conservatives being more reticent to vaccinate than liberals^44^, or divides between supporters of parties that are in the governing coalition or outside of it^14^. Instead, we focus on whether partisan elites take clear positions affirming the social contract and collective responsibility to abide by it, or whether partisan elites question collective responsibility and instead emphasize individual liberty. For example, in Italy, Enrico Letta, then the leader of the *Partito Democratico*, publicly backed vaccine mandates calling them an absolute priority to protect public health and encouraging all Italians to comply. By contrast, the leader of *Fratelli d’Italia* Giorgia Meloni denounced efforts to mandate vaccination as a “regime of terror” and emphasized her and her party’s commitment to defending individual liberty. For a fuller discussion of party positioning, see the Supplementary Information. This leads to the following hypothesis:

H4: Supporters of parties whose leaders openly question vaccine mandates and prioritize individual liberty over collective responsibility in their rhetoric will be more likely to be unvaccinated than supporters of other parties.

Finally, willingness to comply with a vaccination social contract should also be significantly associated with the strength of an individual’s willingness to adhere to collective commitments generally, even in policy areas that are unrelated to vaccination itself. Rather than being shaped solely or even primarily by COVID-specific factors, such as concerns about side effects^45^ or the novel nature of mRNA technology^2^, a social contract perspective suggests that a core factor underlying the decision of many to remain unvaccinated is an innate skepticism of collective commitments and a resistance to moral or legal imperatives to honor them. This leads to a final observable implication of the social contract perspective:

H5: Unvaccinated individuals will be less supportive of honoring other collective commitments in issues unrelated to COVID-19 than vaccinated individuals, *ceteris paribus*.

To test these hypotheses, we conduct a series of analyses at both the aggregate and individual level.

## Results

**Fig. 1:**
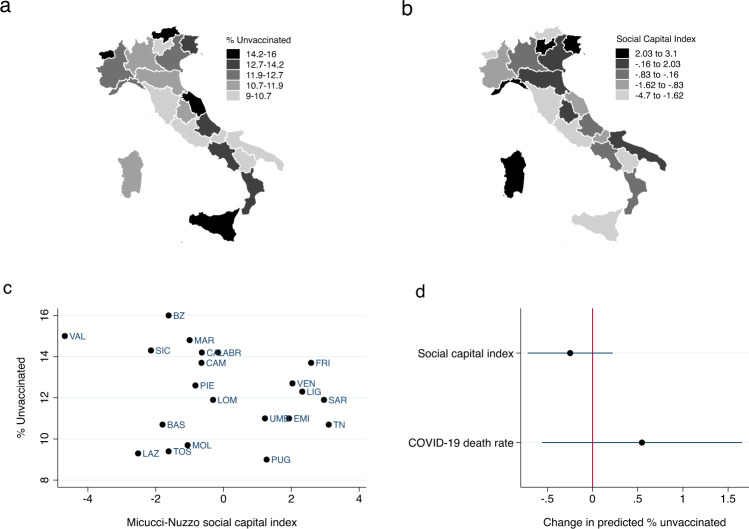
Regional Social Capital and Rate of Unvaccinated Residents. **a** plots the percentage of residents who have not received even a single dose of a COVID-19 vaccine by region. **b** plots an index of macro social capital (capturing civic-mindedness and observance of rules) constructed by Miccucci and Nuzzo. **c** shows the bivariate relationship between regional social capital and the share of population unvaccinated. **d** plots coefficients and 95% confidence intervals from an OLS regression modeling the % unvaccinated as a function of a region’s social capital and COVID-19 death rate.

Despite the best efforts of policymakers to incentivize vaccination, roughly 12% of eligible Italians had not received a COVID-19 vaccine as of May 2022. However, as shown in panel a of Fig. 1, there is considerable variation across regions (the lowest geographic unit of aggregation at which vaccination rate data was available), ranging from a low of 9% in Puglia to a high of 16% in the Autonomous Province of Bolzano.

### Aggregate-level analysis of variation in vaccination rates across regions

To test H1 and examine whether regional variation in social capital correlates with compliance with government mandates to vaccinate against COVID-19, we employ a measure of social capital developed by Miccuci and Nuzzo^46^ specifically designed to capture civic-mindedness (panel b of Fig. 1). The scatter plot in panel c of Fig. 1 shows a weak, but statistically insignificant negative correlation (*r* = −0.23; *p* = 0.31, two-tailed test) between regional social capital and the percent of residents that remain unvaccinated.

An OLS regression modeling the percentage of unvaccinated residents in each region as a function of the region’s social capital level and COVID-19 death rate similarly yields null results (panel d of Fig. 1). Neither factor is a statistically significant predictor of a region’s rate of unvaccinated citizens. Thus, we find little empirical support for H1 that social context, specifically the level of regional social capital, is significantly associated with variation in vaccination rates.

### Individual-level analysis of social trust, rule of law attachments, and vaccination status

To examine factors associated with vaccine behavior at the individual level, we analyze data from an original nationally representative survey of 1000 adult Italians fielded by YouGov from March 14–20, 2022.

We estimate a logistic regression in which the dependent variable is an indicator variable coded 1 for survey respondents who reported not having received any doses of a COVID-19 vaccine and 0 for those who reported receiving one or more doses. The independent variables of interests are a measure of social trust and an index capturing the strength of each individual’s attachment to the rule of law; the model also controls for partisan attachments and demographic factors.

**Fig. 2:**
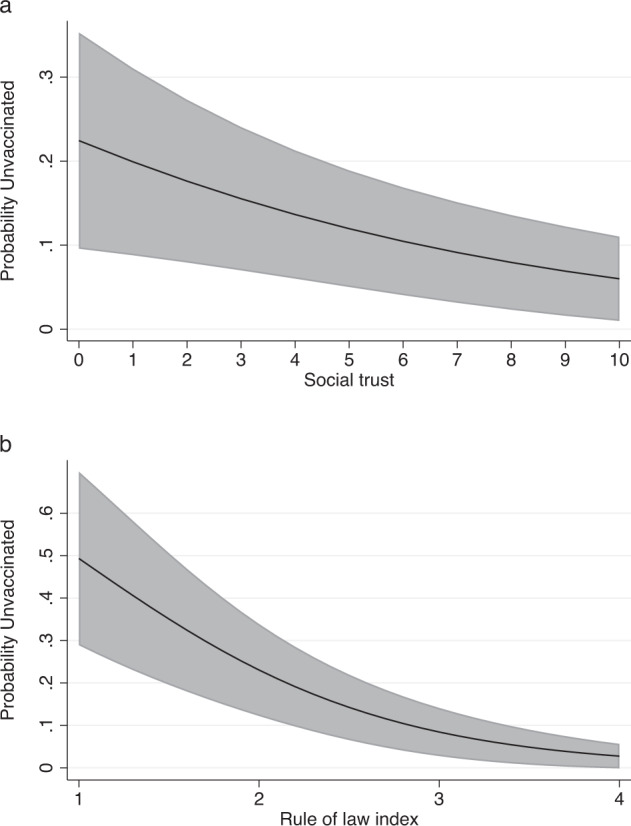
Social Trust, Attachments to the Rule of Law, and Vaccination Status. Predicted probabilities of being unvaccinated by social trust (**a**) and attachments to the rule of law (**b**), holding all other factors constant at their medians. Shaded bands indicate 95% confidence intervals.

While the aggregate-level analysis found little evidence of a relationship between variation in regional social capital and vaccination rates, we do find evidence of a relationship between individual-level variation in social trust and vaccination behaviors. As shown in Fig. 2 (panel a), social trust was inversely and significantly (*p* < 0.01, two-tailed test) associated with the likelihood of being unvaccinated. Moving from the lowest to the highest value on the social trust scale is associated with a reduction in the predicted probability of being unvaccinated from 22% to 6%. These results are consistent with H2.

Strongly consistent with H3, commitments to the rule of law (panel b) had the strongest negative association with being unvaccinated (*p* < 0.001, two-tailed test). Subjects with the weakest commitment to the rule of law in our sample had a 50% predicted probability of being unvaccinated, all other variables held at their median values. By contrast, respondents with the median attachment to the rule of law score had only an 12% predicted probability of being unvaccinated. And for those in the top decile of the rule of law distribution, the predicted probability of being unvaccinated was less than 5%.

### Partisan divides

**Fig. 3:**
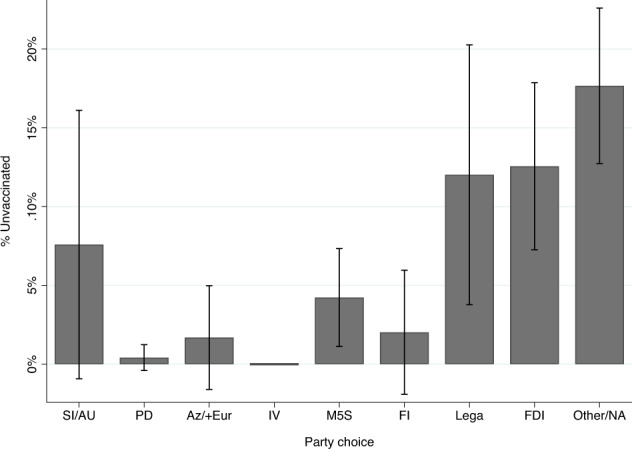
Political Partisanship and Vaccination Status. Bars presents the percentage unvaccinated by supporters of each party. None of the 17 supporters of *Italia Viva* (*IV*) reported being unvaccinated. I-bars present 95% confidence intervals.

To test H4 about partisan divides in vaccination, Fig. 3 plots the percentage of respondents unvaccinated and corresponding 95% confidence intervals for supporters of each major political party. The results are strongly consistent with H4.

Figure 3 shows significant variation in the percentage unvaccinated across partisan groups; however, this variation does not fall completely along ideological lines, nor is the split primarily between supporters of parties within and outside of the governing coalition. There is a significant ideological divide in vaccination among those who backed a major party, with 9.6% of right-wing party supporters (*Lega, Fratelli d’Italia* (*FDI*), and *Forza Italia* (*FI*)) being unvaccinated versus just 1.5% of supporters of left-wing parties (*Partito Democratico (PD*)*; Azione/+Europa; Italia Viva;* and *Sinistra Italiana/Articolo Uno*); this difference in means is statistically significant, *p* < 0.01, two-tailed test. Left-wing party elites were consistently more supportive of government efforts to mandate vaccination (see Supplementary Information).

However, there is also significant variation within the ideological right. Among supporters of *Lega* and *FDI*, whose leaders repeatedly criticized government efforts to mandate vaccination as a threat to individual liberty (see Supplementary Information), 12.4% remained unvaccinated. By contrast, just 2% of *Forza Italia* supporters, another party of the center-right whose leaders were much more aggressively pro-vaccine (see Supplementary Information), reported being unvaccinated (this difference in means is statistically significant, *p* < 0.01, two-tailed test).

This division within the ideological right is strongly consistent with H4. Italians whose trusted co-partisan elites were most critical of government policies to incentivize or mandate vaccination and who emphasized individual liberty over collective responsibility in their rhetoric were significantly more likely to be unvaccinated than either supporters of left-leaning parties or supporters of right-leaning parties whose leaders publicly and consistently supported vaccination. Additional analyses examining the associations between faith in the leaders of the major parties and the likelihood of being unvaccinated yield complementary results (see Supplementary Information).

Finally, vaccine refusal was highest among those who did not back a major political party with 17.7% not having received a single dose. This is significantly higher than the percentage unvaccinated among supporters of parties whose elites publicly supported vaccination policies (17.7% vs. 2.4%; difference in means is statistically significant, *p* < 0.001, two-tailed test). It is also higher than the percentage unvaccinated among supporters of the two parties (*Lega* and *FDI*) whose leaders openly expressed skepticism of government mandates and criticized such policies as a threat to individual liberty (12.4%); however, the difference in means is not statistically significant (17.7% vs. 12.4%, *p* = 0.12, two-tailed test).

### Vaccination status and international commitments

Social contract theories of vaccination emphasize the moral obligation at the heart of the contract for all in society to comply for the public good. If weak commitment to collective moral obligations underlies or enables the unwillingness of many to refuse vaccination, then the unvaccinated should also exhibit less commitment to collective moral obligations in other settings.

To test H5, we examine the associations between being unvaccinated and a series of questions about opinion toward one of the most pressing international issues at the time our survey was in the field: the Russian invasion of Ukraine. For many Europeans, the crisis itself invoked norms of collective obligations under the responsibility to protect doctrine, an argument echoed publicly by Ukrainian President Volodymyr Zelensky in the early days of the invasion^47^. Moreover, the very real concern that the war would spillover across borders into a NATO country directly involved questions of whether citizens would be willing to honor collective commitments and defend an ally.

Specifically, we estimate three logistic regressions in which the independent variable of interest is an indicator variable identifying unvaccinated respondents. All models include partisan attachment indicators and demographic controls. The first model assesses support for the Italian government’s efforts to send both humanitarian and military aid to Ukraine in the aftermath of the Russian invasion. The second assesses concern that the war in Ukraine could trigger a larger nuclear confrontation. The third assesses willingness to send Italian troops to defend a NATO ally should it be attacked by Russia.

**Fig. 4:**
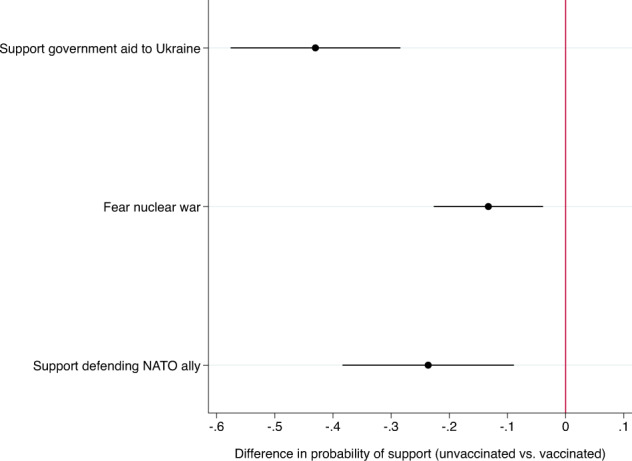
Relationship between Being Unvaccinated and Attitudes toward Ukraine War. *Note:* Marginal effects for indicator variable identifying unvaccinated individuals from three logistic regressions. Each logistic regression also controlled for political partisanship and demographic factors. Horizontal lines present 95% confidence intervals.

Figure 4 presents the difference in predicted probability of answering each question in the affirmative between the median unvaccinated respondent and the median respondent who had received at least one dose of a COVID-19 vaccine, holding all other variables constant. On each question, we observe statistically significant differences in opinions between the vaccinated and the unvaccinated. However, the largest differences are on the first and third questions, which directly involve an imperative to help others and honor collective commitments.

In the first model, unvaccinated respondents were 43% less likely to support government aid to Ukraine than were vaccinated respondents. Unvaccinated respondents were 13% less likely to say they feared the conflict would spill-over into a nuclear war than were vaccinated Italians, all else equal. Finally, unvaccinated respondents were 24% less likely to support sending Italian troops to defend a NATO ally if attacked by Russia. All of these differences in opinions between vaccinated and unvaccinated respondents were observed after controlling for partisan preferences and other demographic factors.

This pattern is strongly consistent with H5. Not only did unvaccinated respondents hold significantly different opinions on these important policy questions than vaccinated respondents, but consistent with theoretical expectations, these opinion gaps were also largest on the questions that explicitly involved collective obligations to help others in the international community and to honor collective commitments.

## Discussion

Governments have embraced an array of policies to incentivize vaccination from barring the unvaccinated from participating in work and many every-day social activities^48^ to mandating vaccination^49^. While such instruments have enjoyed considerable success in boosting vaccination rates, even in countries with stringent mandates, such as Italy, sizable numbers of unvaccinated individuals remain. Understanding the factors underlying this behavior may inform debates over outreach efforts.

Many analyses of vaccine hesitancy have focused on the influence of vaccine and COVID-19 specific factors, such as fears about side effects, concerns about the accelerated development process, and skepticism of vaccines in general^4,50^. These factors are undoubtedly important. However, particularly at this advanced stage of the pandemic and in contexts where governments have aggressively moved to incentivize or even mandate vaccination, other factors, such as variation in underlying commitments to the social contract, may be of paramount importance in explaining who continues to resist vaccination in the face of enormous governmental pressure and why. We find considerable, though not universal support for a social contract perspective suggesting that those who remain unvaccinated in Italy are those most skeptical of collective commitments.

We find little evidence that social context corresponds with regional variation in the ranks of the unvaccinated in Italy. While past research has found that regional social capital reserves are inversely correlated with COVID-19 case and death rates^51,52^ and compliance with public health social distancing guidelines^53^, they are not significantly associated with vaccination uptake. Residents of regions with strong social networks of trust and reciprocity were not more likely to be vaccinated than residents of regions with lower levels of social capital, all else being equal.

By contrast, we find that individuals’ attachments to collective commitments are significantly associated with vaccination status. This affirms insights from a recent study in Germany^54^ that finds social motivations underlie attitudes toward vaccination and extends the focus by looking at the range of collective commitments that are associated with vaccine behavior. Individual-level variation in social trust was significantly associated with vaccination status. High social trust survey respondents were significantly less likely to be unvaccinated, all else equal. This speaks to a debate within the literature about the relationship between vaccine uptake and social trust with some studies finding a positive relationship^55^, others a null relationship^29^, and still others a negative relationship^56^. In the Italian context where the government embraced particularly aggressive policies to incentivize vaccination, social trust was a strong and significant predictor of vaccine uptake.

Support (or lack thereof) for the rule of law, a core common commitment of a democratic society, was even more strongly associated with being unvaccinated. Italians with weak commitment to the rule of law were much more likely to be unvaccinated than those with strong attachments to the rule of law.

Moreover, unvaccinated individuals were much more likely to reject collective responsibility to help others in widely different settings – either when there is a moral imperative to do so, as in the case of aiding Ukraine, or a legal one, as in the case of aiding an ally under attack. We do not argue this association is evidence of a causal relationship. Rather, precisely because we would not otherwise expect to see a correlation between foreign policy opinions and individual vaccination behavior, our results suggest that the same underlying factor – a skepticism toward collective commitments – underlies both resistance to vaccination and a reticence to support aiding Ukraine and a NATO ally if attacked by Russia.

Finally, we find significant partisan divides between supporters of parties whose elites sought to encourage vaccination and bolster the social contract versus supporters of parties whose leaders criticized government mandate policies as threats to individual liberties. The divide within the ideological right is particularly striking; supporters of *Lega* and *Fratelli d’Italia*, whose leaders openly criticized mandates as threats to individual liberty, were more than five times more likely to be unvaccinated than supporters of *Forza Italia*, whose leaders consistently supported efforts to incentivize vaccination^20^. This pattern is consistent with theories of elite opinion leadership^12,39,40,57^. The stark partisan divide also confirms a growing literature suggesting that the politicization of pandemic policies is not a “uniquely American tragedy”^58^ and corroborates recent work pointing to a broader cross-national phenomenon^59^.

The high percentage of unvaccinated respondents among those who did not affiliate with a major political party was also notable and somewhat surprising. Consistent with expectations, the percentage unvaccinated in this group far exceeded that among supporters of parties whose elites rallied behind the government vaccination initiatives. However, the percentage of unvaccinated among those not affiliated with a major party was even higher than among supporters of *Lega* and *Fratelli d’Italia* (though the difference was not statistically significant). There are many reasons individuals may not affiliate strongly with a political party^60^. Not all respondents who did not support a major party in our survey are apolitical or alienated from politics, but many may be. This result suggests that in many contexts, this group may be the most resistant to government policies to incentivize vaccination, and possibly also the most difficult for public health campaigns to reach. This is an important ground for future research.

Previous research has emphasized the importance of COVID-specific beliefs and fears^2^, and trust in institutions directly responsible for shaping pandemic policy^5–7^, in driving resistance to vaccination. Our results suggest that those who remain unvaccinated even after extraordinary government efforts to incentivize it are likely motivated by a deeper skepticism of collective commitments and governing institutions. Given these findings, making the social contract more explicit^18^ and explaining the concept of herd immunity and the social benefit of vaccination^61^, in and of itself, is unlikely to convert the remaining hold-outs. Rather, appeals by groups independent of the state emphasizing individual benefits may be the most effective instrument.

Despite offering valuable empirical study of hold-outs to COVID-19 vaccine mandates, our study has several important limitations. First, the aggregate-level analysis is only able to examine correlations between social capital and vaccination rates at the regional level. While more fine-grained social capital data exists, vaccination data is only available at the regional level. The resulting small number of observations limits the statistical power of our tests; indeed, both the bivariate correlation between regional social capital and the share of the population that remained unvaccinated and the relevant regression coefficients across model specifications (see Supplementary Table 1) are negative, however they fail to reach conventional levels of statistical significance. While our data suggests the relationship between regional social capital and vaccination rates is weaker than the relationships between regional social capital in Italy and other outcomes of interest^52,53^, with our limited data it is possible we are failing to detect a weaker, though still substantively meaningful relationship. Moreover, working at this higher level of geographic aggregation misses important heterogeneity in social context within regions that may influence vaccination decisions. The weak empirical evidence for a relationship found here should spur additional research where more fine-grained data is available.

Second, the relationships uncovered in the analysis of individual-level survey data are strictly associational. For example, while we have argued that the most likely interpretation of the strong inverse relationship between attachment to the rule of law and vaccination status is that the former, which literatures in judicial politics argue is an underlying core value^33,34^, influences the latter, the reverse is also possible. The decision to resist government calls to vaccinate may weaken attachments to the rule of law. We cannot distinguish between these possibilities with a single cross-sectional survey. Thus, we are careful to emphasize that our associational results are consistent with a series of observable implications derived from a social contract perspective of vaccination, even if the data do not allow for causal claims.

Third, while we found evidence of strong, statistically significant partisan divides consistent with our theoretical expectations, future research with larger survey samples could produce more precise estimates of vaccination rates across parties, particularly among supporters of smaller parties.

Fourth, while we have argued that variation in individuals’ attachments to the social contract may offer new insights into who continues to resist COVID-19 vaccine mandates, our study cannot test the relative explanatory power of a social contract perspective versus COVID-19 and vaccine-specific concerns. Our study was not designed to do this, in part, because survey questions measuring general concern with vaccines, fears about side effects, and self-reported willingness to vaccinate against COVID-19 may all be indicators of the same underlying latent dimension^62^. As such, our results do not show that social trust, attachments to the rule of law, and willingness to honor collective commitments are stronger predictors of vaccine uptake than other factors; rather, our results point to the importance of such core underlying concerns in understanding who remains unvaccinated in the face of government mandates.

Finally, our results raise important questions of generalizability. A social contractarian perspective could and should be particularly influential in explaining who remains unvaccinated following a massive government effort to incentivize and even mandate vaccination backed by severe penalties for noncompliance. However, our empirical analysis was restricted to Italy, which had a particularly broad set of vaccine mandates, in March 2022. We urge further study of other countries with COVID-19 vaccine mandates, such as New Zealand, Australia, or Canada, to understand whether the factors associated with hold-outs in Italy have broader generalizability, or whether their explanatory power depends on the type of vaccine mandates imposed by the government. Our results cannot speak directly to contexts where there were not such aggressive government policies to incentivize or mandate vaccination. However, even absent such policies, we believe individuals with weaker attachments to collective commitments, lower levels of social trust, and weaker bonds to the social contract will be more likely to resist vaccination efforts, even if such individuals comprise a smaller overall share of the unvaccinated. This is an important ground for future research. Acknowledging these limitations, we believe our findings offer an important complement to the extensive literature on vaccine hesitancy by emphasizing the importance of broader attitudes toward collective commitments and the social contract in shaping vaccination decisions.

## Methods

To study the relationship between vaccination status and adherence to the social contract, we conduct a pair of analyses at both the aggregate and individual level.

### Aggregate-level data and measures

First, we examine variation in the percentage of Italians unvaccinated across regions and examine whether this correlates with regional measures of social capital and civic mindedness^24^. Data on vaccination rates by region were collected as of May 13, 2022, from the *Commissario Straordinario per l’emergenza Covid-19* (https://github.com/italia/covid19-opendata-vaccini). Data were reported collectively for the region Trentino-Alto Adige. Disaggregated data for the Autonomous Province of Trento and Autonomous Province of Bolzano were taken from Johns Hopkins University’s COVID-19 Data Repository (https://github.com/CSSEGISandData/COVID-19). The combined data was identical across the two sources. To measure social capital, we employ Micucci and Nuzzo’s^46^ measure of macro social capital, which was created to correspond to Putnam’s^24^ emphasis on civic-mindedness and observance of rules. Specifically, we used the measure constructed using a smaller data set eliminating variables shown to be weakly connected to the theoretical literature and that nets out the effect of per capita value added (see pp. 170-172). To construct regional COVID-19 death rates, we use data from regional civil protection reports (https://github.com/pcm-dpc/COVID-19) and population data from ISTAT (https://demo.istat.it/popres/index.php?anno=2022&lingua=ita).

### Aggregate-level analysis

To assess the association between regional social capital and vaccination rates, we first calculate the bivariate correlation coefficient between the two variables across the twenty-one regions. We then estimate an OLS regression modeling the percentage of residents five years of age and older in each region that have not yet received a single dose of a COVID-19 vaccine on the Micucci-Nuzzo social capital index and the COVID-19 death rate in each region (panel d, Fig. 1). Full regression results are presented in model 1 of Supplementary Table 1.

As a robustness check, we also re-estimated the regression analysis using three alternate measures of regional social capital: Cartocci’s^63^ social capital index, which is composed of four items: electoral participation, newspaper readership, the size of the non-profit sector, and blood donations; and Sabatini’s^64^ measures of both bonding and bridging social capital. In each specification, the relevant coefficient for regional social capital is negative; however, in no specification is the coefficient statistically significant (models 2-4 of Supplementary Table 1).

### Individual-level data and measures

To examine the factors associated with identifying as unvaccinated despite extensive government efforts to incentivize vaccination, we fielded a nationally representative survey of 1000 adult Italians with the survey firm YouGov from March 14–20, 2022. Respondents were matched to a sampling frame based on the 2019 Eurobarometer Survey, and were then weighted on post-stratified region, 2018 vote choice, and a three-way stratification based on gender, age, and education. Sample demographics are presented in Supplementary Table 2. Complete question wording for all questions analyzed are provided below. Before beginning the survey, all respondents provided informed consent and were free to stop at any time. All survey protocols were approved by Cornell’s Institutional Review Board, protocol #2007009729.

### Vaccination status

To measure vaccination status subjects were asked: “which of the following best describes your COVID-19 vaccination status?” From this we created an indicator variable coded 1 for those who chose “I have NOT received the COVID-19 vaccine” and 0 for those who said they had received one (2% of sample), two (11% of sample), or three (78% of sample) doses. Twenty-one respondents declined to answer this question and are excluded from the analysis.

### Social trust and rule of law

To measure social trust, we adopt the relevant question from the Global Preference Survey^65^: “I assume that people have only the best intentions.” Respondents indicated on a zero to ten scale how well the statement described them personally.

To measure the strength of individual attachment to the rule of law, we used three survey items validated in cross-national settings^33^: “it is not necessary to obey a law you consider unjust”; “it is not necessary to obey the laws of a government I did not vote for”; and “the government should have some ability to bend the law in order to solve pressing social and political problems”. Respondents evaluated each statement on a four-point scale ranging from strongly agree to strongly disagree. Disagreement with each statement indicated stronger support for the rule of law, and we averaged responses across the three statements to create a four-point support for the rule of law index.

### Partisanship

To measure partisan attachments, we asked respondents which party they would be most likely to vote for if a parliamentary election were held tomorrow. Answer choices included the eight most popular parties according to recent polls and an “other” option. In consultation with YouGov, we chose the eight most popular parties according to *Politico’s* poll of polls (https://www.politico.eu/europe-poll-of-polls/italy/) the week before our survey went into the field. Following *Termometro Politico*, we combined Articolo Uno and Sinistra Italiana. The resulting list of eight parties includes every party with a minister in the Draghi government; the largest opposition party, *Fratelli d’Italia*; and the largest party supporting the government without a minister (*Azione/+Europa*). This includes every party averaging even 3% support (the required threshold to win a parliamentary seat awarded via proportional representation) across polls in the three months before our survey went into the field.

From this question, we constructed a series of eight indicator variables identifying respondents who supported a major party. A ninth indicator identified those who selected “other” or did not choose any party.

### Ukraine and collective international commitments

The survey included three questions measuring opinions toward the war in Ukraine. The first queried general support for the Italian government’s efforts to provide humanitarian and military aid to Ukraine. This was a split sample question. Half of the sample was told: “Since Russia’s invasion of Ukraine, the Italian government has taken steps to increase aid for Ukrainian refugees and allow arms to be sent to Ukraine’s government.” The other half of the sample was told: “Since Russia’s invasion of Ukraine, the Italian government has declared a state of emergency and issued a decree to increase aid for Ukrainian refugees and allow arms to be sent to Ukraine’s government”. All respondents were then asked: “Do you support or oppose these government actions to assist Ukraine?” Response options were a four-point scale ranging from strongly support to strongly oppose. A midpoint was omitted to guard against satisficing^66,67^. Because the percentage supporting a policy is the most important political quantity of interest, we collapsed the strongly and somewhat support categories to create a binary dependent variable coded 1 for those who supported government actions to aid Ukraine and 0 for those who opposed it.

Our second measure of opinions toward Ukraine that involves collective commitments queried support for collective defense among NATO members. This was a split sample question. Half of the sample was asked: “If Russia invades a NATO ally, do you think Italy should send troops to defend that ally against Russian aggression?” The other half of the sample was asked the same question, but was first informed that “Article 5 of the NATO charter states that an attack against any NATO member is an attack against all members of the alliance, including Italy.” Response options were, “yes, should send troops”; “no, should not send troops”; and “don’t know.” From this we created a binary dependent variable coded 1 for those who supported sending troops to defend a NATO ally and 0 for those who did not or did not know.

The final question gauged the degree of concern that the conflict could escalate to a nuclear exchange. All subjects were asked: “Russia’s President Vladimir Putin made reference to the dire consequences facing any country that tries to counter the invasion of Ukraine. Are you concerned that Russia may use nuclear weapons if NATO, which includes Italy, tries to interfere with the invasion, or not?” Response options were, “yes, concerned”; “no, not concerned”; and “don’t know.” From this we created a binary dependent variable coded 1 for those who supported sending troops to defend a NATO ally and 0 for those who did not or did not know.

The three items do not tap a single underlying dimension of support for Ukraine. Inter-item correlations are weak; the strongest is between support for the government’s actions to aid Ukraine and support for defending a NATO ally, but these are only correlated at *r* = 0.33. Cronbach’s alpha for the three items is only 0.34. As a result, we analyze each item separately.

### Individual-level analysis: factors associated with being unvaccinated

To estimate the relationship between social trust, attachments to the rule of law, and being unvaccinated we estimate a logistic regression that also controlled for partisanship and a range of demographic characteristics including gender, educational attainment, and age (for an additional investigation of the relationship between age and vaccinations status, see Supplementary Fig. 5 and Supplementary Table 6). Full regression results are presented in model 1 of Supplementary Table 3. Figure 2 plots marginal effects for each factor holding all other factors constant at their medians.

To analyze the relationship between partisanship and vaccination status, we calculate the means and 95% confidence intervals identifying as unvaccinated across supporters of eight major parties; the final group includes those who selected “other” (*altro*) or who refused to answer the question. Results are presented in Fig. 3.

### Individual-level analysis: associations between being unvaccinated and preferences toward Ukraine

To examine the association between being unvaccinated and each Ukraine-related opinion, we estimate a series of logistic regressions (Supplementary Table 4). In each, the unvaccinated indicator was the independent variable of interest, and each model also controlled for the eight partisan indicators and demographic factors. In the support government aid to Ukraine and defend NATO ally regressions, the models also included an indicator variable identifying assignment to the second wording variant of the split samples. Figure 4 plots the change in predicted probability associated with being unvaccinated vs. vaccinated on all three questions while holding all other variables constant at their median values.

### Robustness checks: different measure of vaccination

The analyses in the text focused on the characteristics of the 9% of Italians who have not received a single dose of a COVID-19 vaccine. However, an additional 2% reported having received one dose of a COVID-19 vaccine. Few Italians received the single-shot Janssen vaccine, and most of those who had received only a single shot at the time of our survey were unlikely to meet the requirements for a super green pass (*rafforzato*), which required subjects to be fully vaccinated and to receive a booster shot within six months of completing the initial course of vaccination. As a robustness check, we re-estimated the analyses in Figs. 2–3 using an alternate measure of non-compliance with government incentives/mandates: an indicator variable coded 1 for those who had received 1 or fewer doses of a COVID-19 vaccine and 0 for those who had received 2 or 3 doses. Results are substantively similar (Supplementary Table 3; Supplementary Figs. 1-2).

### Robustness checks: different measures of partisan attachments

In addition to the party preference measure described previously, we also measured how much faith respondents had in the then-current prime minister (*presidente del consiglio dei ministri*), Mario Draghi, and the leaders of the five largest political parties (Matteo Salvini, Silvio Berlusconi, Giorgia Meloni, Enrico Letta, and Giuseppe Conte) on a four-point scale from no faith (*per nulla/nessuna fiducia*) to a great deal of faith (*molta fiducia*).

To analyze the relationship between being unvaccinated and faith in political leaders, we estimate a series of six OLS regressions, with each regressing faith in a political leader on an indicator variable identifying unvaccinated respondents; the eight partisan indicator variables described above; and demographic characteristics including gender, age, and educational attainment (Supplementary Table 5). Supplementary Fig. 3 illustrates the results. The coefficients and 95% confidence intervals are the predicted differences in faith in each politician between the vaccinated and unvaccinated, all else being equal.

The results reveal similar partisan divides that correspond to those observed in Fig. 3. As would be expected since the Draghi government instituted the most draconian policies to incentivize vaccination, the unvaccinated were least trusting in Draghi compared to other Italians, all else equal. However, they were also significantly less trusting in *Movimento Cinque Stelle* leader and former prime minister Giuseppe Conte, *PD* head Enrico Letta, and long-time leader of *FI* Silvio Berlusconi. The only leader for which unvaccinated Italians did not have significantly less faith than vaccinated Italians is *FDI’s* Giorgia Meloni. The coefficient for faith in *Lega*’s Matteo Salvini is negative and statistically significant, but substantively smaller than for Conte, Letta, or Berlusconi.

### Robustness check: split sample questions

Two of the Ukraine questions employed split samples with slightly different question wordings. The regression analyses whose results are presented in the text account for this by including an indicator variable identifying assignment to either question wording condition. As a robustness check, we estimated alternate models using only data from respondents who received the first wording option in each case; these analyses yield virtually identical results to those reported in Fig. 4 (Supplementary Fig. 4).

### Reporting summary

Further information on research design is available in the Nature Research Reporting Summary linked to this article.

## Supplementary information

Supplementary Materials

REPORTING SUMMARY

## Data Availability

All data and code to replicate the analyses are publicly available at the Harvard Dataverse: 10.7910/DVN/2V4IBP.

## References

[1] Schaffer Deroo S, Pudalov NJ, Fu LY (2020). Planning for a COVID-19 vaccination program. JAMA - J. Am. Med. Assoc..

[2] Rosenbaum L (2021). Escaping catch-22 — Overcoming covid vaccine hesitancy. N. Engl. J. Med..

[3] Luyten J, Bruyneel L, van Hoek AJ (2019). Assessing vaccine hesitancy in the UK population using a generalized vaccine hesitancy survey instrument. Vaccine.

[4] Troiano G, Nardi A (2021). Vaccine hesitancy in the era of COVID-19. Public Health.

[5] Fisher KA (2020). Attitudes toward a potential SARS-CoV-2 vaccine: a survey of U.S. adults. Ann. Intern Med..

[6] Palamenghi L, Barello S, Boccia S, Graffigna G (2020). Mistrust in biomedical research and vaccine hesitancy: the forefront challenge in the battle against COVID-19 in Italy. Eur. J. Epidemiol..

[7] Bollyky, T. J. et al. Pandemic preparedness and COVID-19: an exploratory analysis of infection and fatality rates, and contextual factors associated with preparedness in 177 countries, from Jan 1, 2020 to Sept 30, 2021. *Lancet* **399**, 1489–1512 (2022). 10.1016/S0140-6736(22)00172-6 PMC8806194 35120592

[8] Lazarus JV (2021). A global survey of potential acceptance of a COVID-19 vaccine. Nat. Med.

[9] Khubchandani J (2021). COVID-19 vaccination hesitancy in the United States: a rapid national assessment. J. Community Health.

[10] Gadarian, S. K., Goodman, S. W. & Pepinsky, T. B. *Pandemic politics: the deadly toll of partisanship in the age of COVID*. (Princeton University Press, 2022).

[11] Grossman G, Kim S, Rexer JM, Thirumurthy H (2020). Political partisanship influences behavioral responses to governors’ recommendations for COVID-19 prevention in the United States. Proc. Natl. Acad. Sci. USA.

[12] Pink SL, Chu J, Druckman JN, Rand DG, Willer R (2021). Elite party cues increase vaccination intentions among Republicans. Proc. Natl. Acad. Sci. USA.

[13] Kreps SE, Kriner DL (2021). Factors influencing Covid-19 vaccine acceptance across subgroups in the United States: evidence from a conjoint experiment. Vaccine.

[14] Ward JK (2020). The French public’s attitudes to a future COVID-19 vaccine: the politicization of a public health issue. Soc. Sci. Med..

[15] Karafillakis E, Van Damme P, Hendrickx G, Larson HJ (2022). COVID-19 in Europe: new challenges for addressing vaccine hesitancy. Lancet.

[16] Gostin LO, Salmon DA, Larson HJ (2021). Mandating COVID-19 Vaccines. JAMA - J. Am. Med. Assoc..

[17] Attwell K (2022). COVID-19 vaccine mandates: an Australian attitudinal study. Vaccine.

[18] Korn L, Böhm R, Meier NW, Betsch C (2020). Vaccination as a social contract. Proc. Natl. Acad. Sci. USA.

[19] Weisel O (2021). Vaccination as a social contract: the case of COVID-19 and US political partisanship. Proc. Natl. Acad. Sci. USA.

[20] Profeti S (2022). ‘I hope you like jabbing, too’. The Covid vaccination campaign in Italy and the measures to promote compliance. Contemp. Ital. Politics.

[21] Attwell K, Drislane S, Leask J (2019). Mandatory vaccination and no fault vaccine injury compensation schemes: an identification of country-level policies. Vaccine.

[22] Stefanizzi P, Bianchi FP, Brescia N, Ferorelli D, Tafuri S (2022). Vaccination strategies between compulsion and incentives. The Italian Green Pass experience. Expert Rev. Vaccines.

[23] Wilf-Miron R, Myers V, Saban M (2021). Incentivizing vaccination uptake: The “Green Pass” proposal in Israel. JAMA.

[24] Putnam, R. D. *Making Democracy Work: Civic Traditions in Modern Italy*. (Princeton University Press, 1993).

[25] Bowles S, Gintis H (2002). Social capital and community governance. Econ. J..

[26] Whiteley PF (2000). Economic growth and social capital. Polit. Stud. (Oxf.).

[27] Shortt SED (2004). Making sense of social capital, health and policy. Health Policy (N. Y.).

[28] Faezi NA (2021). Peoples’ attitude toward COVID-19 vaccine, acceptance, and social trust among African and Middle East countries. Health Promot Perspect..

[29] Jennings W (2021). Lack of trust, conspiracy beliefs, and social media use predict COVID-19 vaccine hesitancy. Vaccines (Basel).

[30] Roy DN, Biswas M, Islam E, Azam MS (2022). Potential factors influencing COVID-19 vaccine acceptance and hesitancy: a systematic review. PLoS ONE.

[31] Böhm R, Betsch C, Korn L (2016). Selfish-rational non-vaccination: experimental evidence from an interactive vaccination game. J. Econ. Behav. Organ.

[32] Hershey JC, Asch DA, Thumasathit T, Meszaros J, Waters VV (1994). The roles of altruism, free riding, and bandwagoning in vaccination decisions. Organ Behav. Hum. Decis. Process.

[33] Gibson JL (2007). The legitimacy of the U.S. supreme court in a polarized polity. J. Empir. Leg. Stud..

[34] Reeves A, Rogowski JC (2016). Unilateral powers, public opinion, and the presidency. J. Polit..

[35] Christenson DP, Kriner DL (2017). The specter of supreme court criticism: public opinion and unilateral action. Pres. Stud. Q.

[36] Gibson JL (2007). Changes in American veneration for the rule of law. DePaul Law Rev..

[37] Clayton K (2021). Elite rhetoric can undermine democratic norms. Proc. Natl Acad. Sci. USA.

[38] Green, D., Palmquist, B. & Schickler, E. *Partisan hearts and minds: political parties and the social identities of voters*. (Yale University Press, 2002).

[39] Zaller, J. *The nature and origins of mass opinion*. (Cambridge University Press, 1992).

[40] Bokemper SE, Huber GA, Gerber AS, James EK, Omer SB (2021). Timing of COVID-19 vaccine approval and endorsement by public figures. Vaccine.

[41] Broockman DE, Butler DM (2017). The causal effects of elite position-taking on voter attitudes: field experiments with elite communication. Am. J. Pol. Sci..

[42] Gadarian SK, Goodman SW, Pepinsky TB (2021). Partisanship, health behavior, and policy attitudes in the early stages of the COVID-19 pandemic. PLoS One.

[43] Argote P (2021). The shot, the message, and the messenger: COVID-19 vaccine acceptance in Latin America. NPJ Vaccines.

[44] El-Mohandes A (2021). COVID-19 vaccine acceptance among adults in four major US metropolitan areas and nationwide. Sci. Rep..

[45] Rief W (2021). Fear of adverse effects and COVID-19 vaccine hesitancy: recommendations of the treatment expectation expert group. JAMA Health Forum.

[46] Micucci, G. & Nuzzo, G. Measuring social capital: evidence from Italy. *SSRN Electronic J.*10.2139/ssrn.2160456 (2012).

[47] Bosse G (2022). Values, rights, and changing interests: The EU’s response to the war against Ukraine and the responsibility to protect Europeans. Contemp. Secur. Policy.

[48] Wilf-Miron R, Myers V, Saban M (2021). Incentivizing vaccination uptake: The ‘green Pass’ proposal in Israel. J. Am. Med Assoc..

[49] Burki T (2022). COVID-19 vaccine mandates in Europe. Lancet Infect. Dis..

[50] Lin C, Tu P, Beitsch LM (2021). Confidence and receptivity for covid-19 vaccines: a rapid systematic review. Vaccines.

[51] Bartscher AK, Seitz S, Siegloch S, Slotwinski M, Wehrhöfer N (2021). Social capital and the spread of covid-19: Insights from european countries. J. Health Econ..

[52] Fraser T, Aldrich DP, Page-Tan C (2021). Bowling alone or distancing together? The role of social capital in excess death rates from COVID19. Soc. Sci. Med..

[53] Borgonovi F, Andrieu E (2020). Bowling together by bowling alone: social capital and COVID-19. Soc. Sci. Med..

[54] Schmelz K, Bowles S (2022). Opposition to voluntary and mandated COVID-19 vaccination as a dynamic process: Evidence and policy implications of changing beliefs. Proc. Natl. Acad. Sci. USA.

[55] Dolman, A. J., Fraser, T., Panagopoulos, C., Aldrich, D. P. & Kim, D. Opposing views: associations of political polarization, political party affiliation, and social trust with COVID-19 vaccination intent and receipt. *J. Public Health (Oxf)*10.1093/PUBMED/FDAB401 (2022). 10.1093/pubmed/fdab401 PMC9383304 35077546

[56] Viskupič F, Wiltse DL, Meyer BA (2022). Trust in physicians and trust in government predict COVID-19 vaccine uptake. Soc. Sci. Q.

[57] Chong D, Druckman JN (2007). Framing public opinion in competitive democracies. Am. Political Sci. Rev..

[58] Flores A (2022). Politicians polarize and experts depolarize public support for COVID-19 management policies across countries. Proc. Natl. Acad. Sci. USA.

[59] Becher M, Stegmueller D, Brouard S, Kerrouche E (2021). Ideology and compliance with health guidelines during the COVID-19 pandemic: a comparative perspective. Soc. Sci. Q.

[60] Hajnal, Z. & Lee, T. *Why Americans don’t join the party*. (Princeton University Press, 2011).

[61] Betsch C, Böhm R, Korn L, Holtmann C (2017). On the benefits of explaining herd immunity in vaccine advocacy. Nat. Hum. Behav..

[62] Berinsky AJ, Druckman JN (2007). The polls - review: public opinion research and support for the Iraq war. Public Opin. Q.

[63] Cartocci, R. & Vanelli, V. Una mappa del capitale sociale e della cultura civica in Italia. in *L’Italia e le sue regioni. L’eta repubblicana*. 17–35 (Societa Istituto Enciclopedia Italiana Treccani, 2015).

[64] Sabatini, F. *Measuring social capital in Italy:* an exploratory analysis. unive*rsity of Bologna, faculty of economics, third sector and civil economy working paper series 12* (2005).

[65] Falk A (2018). Global evidence on economic preferences. Q. J. Econ..

[66] Chyung SYY, Roberts K, Swanson I, Hankinson A (2017). Evidence-based survey design: the use of a midpoint on the likert scale. Perform. Improv.

[67] Matell MS, Jacoby J (1972). Is there an optimal number of alternatives for likert scale items? Effects of testing time and scale properties. J. Appl. Psychol..

