# Supplementary material for: Resistance to COVID-19 vaccination and the social contract: evidence from Italy
Source: NPJ Vaccines. 2023 Apr 22;8:60. doi: 10.1038/s41541-023-00660-8 (PMC10122449; doi:10.1038/s41541-023-00660-8)
Supplement: Supplementary file 1 — Supplementary Materials [file 41541_2023_660_MOESM1_ESM.docx]

Supplementary Materials for

**Resistance to COVID-19 Vaccination and the Social Contract: Evidence from Italy**

Sarah E. Kreps and Douglas L. Kriner^*^

*Corresponding author. Email: [kriner@cornell.edu](mailto:kriner@cornell.edu)

**This file includes:**

Supplementary Discussion

Supplementary Figures 1 to 5

Supplementary Tables 1 to 6

Supplementary Discussion

**Partisan Position-taking**

Our argument about vaccination and the social contract expects individuals from parties that have cast the vaccine as government overreach and emphasized individual liberty over collective responsibility will not view vaccination as a social contract and be less likely to vaccinate. Here we offer context on the position of Italian political parties toward government policies that mandate or incentivize COVID-19 vaccination.

The major political parties of the ideological left in Italy have been consistently supportive of efforts to incentivize and even mandate vaccination against COVID-19. The largest party of the left, the *Partito Democratico* (*PD*), has consistently supported stringent public health measures. In December 2021, *PD* leader Enrico Letta publicly backed the government’s extreme efforts to incentivize vaccination and declared that it was time to prepare the next steps, including vaccine mandates.^[[1]](#footnote-1)^ And former prime minister (then also the leader of the *PD*) and current leader of *Italia Viva*, Matteo Renzi, launched an online petition calling for a COVID-19 vaccine mandate.^[[2]](#footnote-2)^ Roberto Speranza, the leader of *Articolo Uno*, has spearheaded the government’s efforts, including the introduction of the “super” green pass (*rafforzato*) and the vaccine mandate for those fifty and older, as Health Minister under both the second Conte and Draghi governments.^[[3]](#footnote-3)^ And in the midst of the Omicron wave, *Azione* leader Carlo Calenda minced few words on Twitter, writing that “tolerating No Vaxxers with 3% of COVID-19 hospitalizations being children under 5 years of age is not only wrong, but immoral. The circulation of the virus must be reduced as much as possible. Even if the vaccine doesn’t exclude the possibility of becoming infected, it makes it less probable. Enough is enough. Zero tolerance.”^[[4]](#footnote-4)^

Two of the main parties of the ideological right, *Lega* and *Fratelli d’Italia* (*FDI*) took much more critical views of vaccine mandates and other measures to incentivize vaccination, often highlighting the cost to individual liberty. As Italy considered expanding vaccine mandates in fall 2021, *FDI* leader Giorgia Meloni likened vaccine mandates to “a regime of terror.”^[[5]](#footnote-5)^ Meloni was no less hyperbolic in her criticism of measures short of vaccine mandates, such as the green pass and “super” green pass (*rafforzato*) to incentivize vaccination by requiring either proof of vaccination or a negative test (not valid for the *rafforzato*) to participate in a wide range of social and economic activities. In August 2021, Meloni denounced the introduction of the green pass, tweeting: “The idea of having to use this Green Pass to be able to participate in communal life is chilling, and the final step towards the realization of an Orwellian society.” Meloni explicitly prioritized individual liberty over communal responsibility, concluding: “For us [i.e. *FDI*] individual liberty is sacred and inviolable.”^[[6]](#footnote-6)^ Similarly, in December 2021, Meloni sharply criticized the super green pass, saying it had failed to slow the spread of the virus and calling it a repression of liberties.^[[7]](#footnote-7)^

Not to be outflanked on the right, *Lega*’s Matteo Salvini, who has a long history of opposing vaccines (in 2018, he called vaccine mandates for school children “useless and in many cases dangerous”^[[8]](#footnote-8)^) and who previously opposed COVID-19 vaccination mandates for teachers, announced that while *Lega* would remain in the government, he would oppose and vote against efforts to extend vaccine mandates.^[[9]](#footnote-9)^ In August 2021, Salvini called on older Italians (over 50) to be vaccinated to protect themselves, but pledged to vote against any vaccine mandate. “I am against any type of mandate, fine, or compulsion; I am always for liberty, even for liberty of care.”^[[10]](#footnote-10)^ And in social media posts, Salvini repeatedly emphasized his opposition to mandates and support for freedom of choice.^[[11]](#footnote-11)^ In fall 2021, he publicly opposed efforts to extend the green pass, stating: “The position of the League is clear: we are for defending the health of citizens, including in the workplace. But you can’t think of extending the green pass obligation to 60 million Italians.”^[[12]](#footnote-12)^ Throughout the pandemic, Salvini has walked a political tightrope given the stark divisions within his own party, playing to anti-vax and anti-green pass sentiment, while eventually compromising and/or failing to block most measures.^[[13]](#footnote-13)^

The main outlier within the ideological right-wing coalition is Silvio Berlusconi’s *Forza Italia* (*FI*). Throughout the pandemic, leaders of *FI* differentiated themselves from *Lega* and *Fratelli d’Italia* by their strong and consistent support for measures to incentivize and even mandate vaccination. As early as July 2021, *FI*’s Renata Brunetta, the Minister for Public Administration in the Draghi Government, declared Salvini’s opposition to vaccine mandates for teachers wrong and said she would go even further, supporting mandates for most public service employees.^[[14]](#footnote-14)^ Similarly, in November 2021 *FI*’s Antonio Tajani publicly supported the introduction of the super green pass which would be available only to vaccinated individuals (and not to those with a negative test) and to extend vaccination mandates to help brake the growth of cases and prevent new closers and lockdowns.^[[15]](#footnote-15)^

Finally, the anti-establishment *Movimento Cinque Stelle* (*M5S*) has often split on questions concerning the green pass and vaccine mandates, reflecting the significant divides throughout *M5S* on most issues.^[[16]](#footnote-16)^

**The Relationship between Age and Vaccination**

Although not a theoretical focus of our study, our data allows us to examine several interesting questions regarding the relationship between age and vaccine uptake. In Italy at the time of our survey, the vaccine was mandatory for all Italians 50 and older. It was not mandatory for Italians under the age of 50; however, all adults were required to show proof of vaccination to obtain the “super” green pass (*rafforzato*) which was required to go to work, ride public transit, enter a bar, and participate in almost every aspect of social life. Figure S5 shows the distribution of our sample by age.

We first conducted a simple difference in means test to compare the percentage of Italians subject to the mandate (i.e. 50 and older) who remained unvaccinated in our sample vs. the percentage of Italians who were not subject to the mandate. The difference was substantively small and statistically insignificant. This is at least suggestive evidence that the formal mandate had little additive effect in incentivizing vaccination above and beyond the very strong incentives provided by the requirements for the “super” green pass.

We also re-estimated earlier analyses (Table S3) with different operationalizations of age brackets. We first re-estimated our main regression with two age dummy variables: one indicating Italians 50-69; and the second Italians 70+ (the top 10% of the sample age distribution). In this regression, Italians under 50 (i.e. those not subject to the mandate) are the omitted baseline category. Results are presented in model 1 of Table S6. The coefficient for Italians aged 50-69 is substantively small and statistically insignificant. The formal mandate did not make Italians in this age bracket more likely to vaccinate than Italian adults under 50 who were only subject to the green pass requirements. The coefficient for Italians 70 and up is negative, suggesting they are less likely than younger Italians under 50 to remain unvaccinated; however, the coefficient is not statistically significant. In model 2, we disaggregated the age distribution further adding another categorical variable for Italians aged 30-49 (making the omitted baseline category Italians under the age of 30). In this model, we again see no evidence of statistically significant differences in vaccination rates across age groups.

**Supplementary Figure 1:** **Social Trust, Attachments to the Rule of Law and Having Received 1 or Fewer Doses.**

*Note:* Predicted probabilities of having received 0 or 1 doses of a COVID-19 vaccine by social trust (panel **a**) and attachments to the rule of law (panel **b**), holding all other factors constant at their medians.

**Supplementary Figure 2: Partisan Differences in Noncompliance (1 or 0 Doses)**

*Note:* Bars presents the percentage with 1 or fewer doses by supporters of each party. I-bars present 95% confidence intervals.

**Supplementary Figure 3: Faith in Leaders**

Note: Markers plot coefficients for an indicator variable identifying respondents who have received 1 or 0 doses of a COVID-19 vaccine from a series of OLS regressions analyzing faith in each leader on a 4-point scale. All models also control for partisanship and demographics.

**Figure S4: Using only “Control” Group of Split Sample Questions**

**Supplementary Figure 4: Using only “Control” Group of Split Sample Questions**

*Note:* Marginal effects for indicator variable identifying unvaccinated individuals from two logistic regressions. Each logistic regression also controlled for political partisanship and demographic factors.

**Supplementary Figure 5: Distribution of Survey Sample by Age**

**Supplementary Table 1: Social Capital, COVID-19 Death Rates, and the Percentage Unvaccinated**

|  | (1) | (2) | (3) | (4) |
| --- | --- | --- | --- | --- |
|  |  |  |  |  |
|  |  |  |  |  |
| Covid death rate per 1,000 | 0.546 | 0.688 | 1.263 | 0.408 |
|  | (0.527) | (0.693) | (0.853) | (0.678) |
| Micucci-Nuzzo social capital index | -0.248 |  |  |  |
|  | (0.223) |  |  |  |
| Cartocci social capital index |  | -0.010 |  |  |
|  |  | (0.025) |  |  |
| Sabatini bonding social capital index |  |  | -0.264 |  |
|  |  |  | (0.234) |  |
| Sabatini bridging social capital index |  |  |  | -0.056 |
|  |  |  |  | (0.231) |
| Constant | 10.799*** | 11.494*** | 8.892*** | 11.180*** |
|  | (1.491) | (2.048) | (2.339) | (1.860) |
|  |  |  |  |  |
| Observations | 21 | 21 | 21 | 21 |
| R-squared | 0.107 | 0.055 | 0.109 | 0.049 |

*Note:* All models are OLS regressions. The dependent variable is the percentage of residents in each region five and older who have not yet received a single dose of a vaccine against COVID-19. Standard errors in parentheses. All significance tests are two-tailed.

* p < .10

** p < .05

*** p < .01

**Supplementary Table 2: Survey Demographics**

| Characteristic | N = 1,000 |
| --- | --- |
| Gender, n (%) |  |
| Female | 509 (51) |
| Male | 491 (49) |
| Age, n (%) |  |
| 18-24 | 75 (8) |
| 25-34 | 152 (15) |
| 35-44 | 158 (16) |
| 45-54 | 228 (23) |
| 55+ | 387 (39) |
| Educational Attainment, n (%) |  |
| No diploma | 6 (1) |
| Elementary school diploma | 23(2) |
| Middle school diploma | 174(18) |
| High school diploma | 372(37) |
| Apprentice/Technical Institute | 119(12) |
| Bachelor’s degree | 96 (10) |
| Masters degree or higher | 203 (20) |
| Household Income (EUR), n (%) |  |
| <25,000 | 408 (50) |
| 25,000 – 49,999 | 290 (36) |
| 50,000 - 99,999 | 93 (11) |
| 100,000< | 20 (2) |
| Contemporary Party Preference, n (%) |  |
| Movimento 5 Stelle | 153(17) |
| Lega | 65(7) |
| Forza Italia | 57(6) |
| Fratelli d’Italia | 158(17) |
| Partito Democratico | 183(20) |
| Azione/+Europa | 57(6) |
| Sinistra Italiana/Articolo Uno | 32 (3) |
| Italia Viva | 17(2) |
| Other (*Altro*) | 205(22) |
| Work Type, n (%) |  |
| Working full time | 388(39) |
| Working part time | 117 (12) |
| Student | 93(9) |
| Retired | 197(20) |
| Unemployed | 91(9) |
| Not working for other reason | 114(11) |

Note: Sample recruited through YouGov from March 14-20, 2022. Totals may not always sum to 100% due to rounding.

**Supplementary Table 3: Factors Associated with Vaccination Status**

|  | (1) | (2) | (3) |
| --- | --- | --- | --- |
|  | Zero doses | One or fewer doses | # of doses |
|  |  |  |  |
| Rule of law index | -1.179*** | -1.214*** | 0.354*** |
|  | (0.245) | (0.222) | (0.058) |
| Social trust | -0.151*** | -0.138*** | 0.040*** |
|  | (0.049) | (0.044) | (0.012) |
| Fratelli d'Italia | -0.692** | -0.420 | 0.246** |
|  | (0.330) | (0.293) | (0.120) |
| Lega | -0.874* | -1.022** | 0.356** |
|  | (0.463) | (0.461) | (0.149) |
| Forza Italia | -2.537** | -1.917*** | 0.571*** |
|  | (1.060) | (0.668) | (0.118) |
| Movimento 5 Stelle | -1.671*** | -1.322*** | 0.475*** |
|  | (0.462) | (0.394) | (0.103) |
| Partito Democratico | -3.743*** | -2.794*** | 0.607*** |
|  | (1.028) | (0.621) | (0.090) |
| Azione/+Europa | -2.518*** | -2.316*** | 0.613*** |
|  | (0.908) | (0.707) | (0.104) |
| Sinistra Italiana/Articolo Uno | -0.928 | -1.053 | 0.435*** |
|  | (0.652) | (0.646) | (0.156) |
| Italia Viva |  | -1.944* | 0.553*** |
|  |  | (1.159) | (0.162) |
| Education | 0.029 | 0.044 | -0.004 |
|  | (0.050) | (0.044) | (0.011) |
| Female | -0.128 | -0.185 | 0.053 |
|  | (0.262) | (0.237) | (0.056) |
| Age: 25-34 | -0.550 | -0.238 | -0.122 |
|  | (0.715) | (0.583) | (0.130) |
| Age: 35-44 | 0.050 | -0.185 | -0.063 |
|  | (0.714) | (0.588) | (0.143) |
| Age:45-54 | 0.341 | 0.136 | -0.190 |
|  | (0.648) | (0.539) | (0.132) |
| Age: 55+ | -0.149 | -0.309 | 0.029 |
|  | (0.641) | (0.523) | (0.124) |
| Constant | 2.070** | 2.337*** | 1.148*** |
|  | (0.925) | (0.785) | (0.226) |
|  |  |  |  |
| Observations | 889 | 906 | 906 |

*Note:* Models 1 and 2 are logistic regressions; model 3 is OLS. In model 1, dependent variable is not having received a single dose of COVID-19 vaccine. In model 2, dependent variable is having received 0 or 1 doses of a COVID-19 vaccine. In model 3, dependent variable is number of vaccine doses received (from 0 to 3). Because all 17 respondents who support *Italia Viva* had received at least one dose, they are excluded from model 1. Standard errors in parentheses. All significance tests are two-tailed.

* p < .10

** p < .05

*** p < .01

**Supplementary Table 4: Vaccination Status and International Commitments**

|  | (1) | (2) | (3) |
| --- | --- | --- | --- |
|  | Support Ukraine aid | Fear nuclear war | Defend NATO ally |
|  |  |  |  |
| Unvaccinated against COVID-19 | -2.033*** | -1.116*** | -1.463*** |
|  | (0.302) | (0.289) | (0.351) |
| Fratelli d'Italia | 0.476* | 0.269 | 0.691*** |
|  | (0.251) | (0.276) | (0.260) |
| Lega | 1.531*** | -0.044 | 0.660* |
|  | (0.376) | (0.375) | (0.342) |
| Forza Italia | 0.780** | -0.596 | 0.319 |
|  | (0.362) | (0.392) | (0.363) |
| Movimento 5 Stelle | 0.885*** | 0.239 | 0.081 |
|  | (0.247) | (0.294) | (0.255) |
| Partito Democratico | 1.363*** | 0.269 | 0.815*** |
|  | (0.266) | (0.287) | (0.242) |
| Azione/+Europa | 1.491*** | -0.179 | 0.951*** |
|  | (0.449) | (0.377) | (0.354) |
| Sinistra Italiana/Articolo Uno | 0.243 | 0.429 | 0.648 |
|  | (0.422) | (0.520) | (0.425) |
| Italia Viva | 0.759 | -1.104* | 0.786 |
|  | (0.639) | (0.594) | (0.580) |
| Education | 0.022 | 0.029 | 0.001 |
|  | (0.033) | (0.037) | (0.031) |
| Female | -0.171 | 0.968*** | -1.205*** |
|  | (0.165) | (0.184) | (0.157) |
| Age: 25-34 | -0.527 | -0.284 | -0.285 |
|  | (0.430) | (0.430) | (0.394) |
| Age: 35-44 | -0.810** | -0.010 | 0.072 |
|  | (0.405) | (0.442) | (0.381) |
| Age:45-54 | -0.847** | -0.077 | -0.025 |
|  | (0.394) | (0.405) | (0.363) |
| Age: 55+ | -0.586 | -0.217 | 0.171 |
|  | (0.379) | (0.387) | (0.346) |
| Constant | 0.864** | 0.852* | -0.067 |
|  | (0.420) | (0.456) | (0.386) |
|  |  |  |  |
| Observations | 906 | 906 | 906 |

*Note:* All models are logistic regressions. Standard errors in parentheses. All significance tests are two-tailed.

* p < .10

** p < .05

*** p < .01

**Supplementary Table 5: Faith in Political Leaders**

|  | (1) | (2) | (3) | (4) | (5) | (6) |
| --- | --- | --- | --- | --- | --- | --- |
|  | Draghi | Conte | Letta | Berlusconi | Salvini | Meloni |
|  |  |  |  |  |  |  |
| Unvaccinated | -1.205*** | -0.365*** | -0.394*** | -0.332*** | -0.177** | 0.145 |
|  | (0.079) | (0.095) | (0.066) | (0.107) | (0.082) | (0.131) |
| Fratelli d'Italia | 0.119 | -0.147 | -0.254*** | 0.729*** | 0.690*** | 1.613*** |
|  | (0.108) | (0.097) | (0.075) | (0.097) | (0.084) | (0.097) |
| Lega | 0.655*** | 0.138 | 0.055 | 0.741*** | 1.490*** | 1.288*** |
|  | (0.139) | (0.155) | (0.125) | (0.131) | (0.113) | (0.113) |
| Forza Italia | 0.635*** | -0.174 | -0.059 | 1.425*** | 0.697*** | 0.878*** |
|  | (0.151) | (0.148) | (0.109) | (0.134) | (0.149) | (0.123) |
| Movimento 5 Stelle | 0.157 | 1.395*** | 0.241*** | -0.101 | -0.070 | -0.130 |
|  | (0.110) | (0.089) | (0.082) | (0.083) | (0.073) | (0.080) |
| Partito Democratico | 0.958*** | 0.643*** | 1.047*** | -0.160** | -0.259*** | -0.302*** |
|  | (0.101) | (0.089) | (0.081) | (0.074) | (0.061) | (0.071) |
| Azione/+Europa | 0.754*** | 0.504*** | 0.730*** | -0.150 | -0.311*** | -0.253** |
|  | (0.142) | (0.162) | (0.126) | (0.098) | (0.065) | (0.099) |
| Sinistra Italiana/Art. Uno | 0.244 | 0.654*** | 0.454*** | -0.038 | -0.188* | -0.358*** |
|  | (0.177) | (0.165) | (0.148) | (0.152) | (0.108) | (0.109) |
| Italia Viva | 0.837*** | -0.394** | 0.356* | 0.846*** | 0.019 | 0.123 |
|  | (0.234) | (0.194) | (0.185) | (0.312) | (0.153) | (0.197) |
| Education | 0.038*** | -0.030** | 0.022** | -0.015 | -0.009 | 0.002 |
|  | (0.012) | (0.011) | (0.010) | (0.011) | (0.009) | (0.010) |
| Female | -0.072 | 0.177*** | -0.018 | -0.088 | -0.004 | -0.038 |
|  | (0.061) | (0.058) | (0.050) | (0.054) | (0.047) | (0.051) |
| Age: 25-34 | -0.096 | -0.278* | 0.059 | -0.067 | -0.101 | 0.158 |
|  | (0.160) | (0.145) | (0.124) | (0.152) | (0.114) | (0.112) |
| Age: 35-44 | -0.277* | -0.223 | 0.050 | -0.063 | 0.035 | 0.262** |
|  | (0.151) | (0.141) | (0.117) | (0.156) | (0.118) | (0.124) |
| Age:45-54 | -0.079 | -0.324** | 0.173 | -0.053 | 0.018 | 0.326*** |
|  | (0.145) | (0.133) | (0.116) | (0.145) | (0.106) | (0.108) |
| Age: 55+ | 0.252* | -0.437*** | 0.291*** | -0.034 | 0.089 | 0.374*** |
|  | (0.141) | (0.130) | (0.109) | (0.141) | (0.103) | (0.104) |
| Constant | 2.112*** | 2.285*** | 1.392*** | 1.684*** | 1.447*** | 1.322*** |
|  | (0.160) | (0.145) | (0.121) | (0.153) | (0.112) | (0.118) |
|  |  |  |  |  |  |  |
| Observations | 906 | 906 | 906 | 906 | 906 | 906 |
| R-squared | 0.322 | 0.363 | 0.323 | 0.332 | 0.389 | 0.541 |

*Note:* All models are OLS regressions. Faith in each leader measured on a four-point scale. Standard errors in parentheses. All significance tests are two-tailed.

* p < .10

** p < .05

*** p < .01

**Supplementary Table 6: The Relationship between Remaining Unvaccinated and Age**

|  | (1) | (2) |
| --- | --- | --- |
|  |  |  |
|  |  |  |
| Rule of law index | -1.226*** | -1.199*** |
|  | (0.243) | (0.242) |
| Social trust | -0.141*** | -0.141*** |
|  | (0.049) | (0.049) |
| Fratelli d'Italia | -0.684** | -0.675** |
|  | (0.328) | (0.327) |
| Lega | -0.877* | -0.885* |
|  | (0.475) | (0.477) |
| Forza Italia | -2.386** | -2.378** |
|  | (1.067) | (1.077) |
| Movimento 5 Stelle | -1.745*** | -1.731*** |
|  | (0.466) | (0.463) |
| Partito Democratico | -3.705*** | -3.653*** |
|  | (1.026) | (1.024) |
| Azione/+Europa | -2.512*** | -2.441*** |
|  | (0.928) | (0.920) |
| Sinistra Italiana/Articolo Uno | -0.906 | -0.876 |
|  | (0.675) | (0.681) |
| Education | 0.023 | 0.019 |
|  | (0.050) | (0.049) |
| Female | -0.112 | -0.110 |
|  | (0.262) | (0.263) |
| Age: 30-49 |  | 0.525 |
|  |  | (0.495) |
| Age: 50-69 | 0.047 | 0.456 |
|  | (0.293) | (0.504) |
| Age: 70+ | -0.944 | -0.537 |
|  | (0.582) | (0.718) |
| Constant | 2.204*** | 1.746** |
|  | (0.678) | (0.828) |
|  |  |  |
| Observations | 889 | 889 |

*Note:* Both models are logistic regressions. In model 1, the omitted baseline age category is adults under the age of 50. In model 2, the baseline age category is adults under the age of 30. Because all 17 respondents who support *Italia Viva* had received at least one dose, they are excluded from the models. Standard errors in parentheses. All significance tests are two-tailed.

* p < .10

** p < .05

*** p < .01

1. Stefano Cappellini. “PD, Letta: ‘Sui Vaccini e’ l’ora dell’obbligo. Senza unita’ sul Quirinale il governo cadrebbe.” *La Repubblica*, December 30, 2021, <https://www.repubblica.it/politica/2021/12/30/news/quirinale_intervista_a_enrico_letta-332065984/?ref=RHTP-VS-I287409039-P8-S1-T1>. [↑](#footnote-ref-1)
2. “Covid, Italia Viva Lancia Una Petizione sull’Obbligo del Vaccine. ‘I No Vax Ostacolano l’Usicta dall’Emergenza.” *La Repubblica*, December 30, 2021, <https://www.repubblica.it/politica/2021/12/30/news/green_pass_italia_viva_petizione_obbligo_vaccino-332156041/>. [↑](#footnote-ref-2)
3. Monica Guerzoni, “Spearnza: ‘Piu Vaccini o si Rischiano Chiusre in Autunno.” *Corriere della Sera*, September 5, 2021, <https://www.corriere.it/politica/21_settembre_05/nuove-chiusure-covid-speranza-obbligo-vaccinale-9848a65e-0dbf-11ec-94b3-ee97ec98a47b.shtml>. [↑](#footnote-ref-3)
4. <https://twitter.com/carlocalenda/status/1480484894270763014>. [↑](#footnote-ref-4)
5. Hannah Roberts. “Italy Gets Tough on the Unvaccinated, Raising Ethics Questions.” *Politico*.com, September 15, 2021, <https://www.politico.eu/article/italy-mulls-mandatory-coronavirus-vaccines-human-rights-debate/>. [↑](#footnote-ref-5)
6. <https://twitter.com/giorgiameloni/status/1414883674282242062>. [↑](#footnote-ref-6)
7. “Coivd, il PD in Pressing Sull’Obbligo Vaccinale. Meloni Attacca il Governo: ‘Reprime la Liberta’.” *La Repubblica,* December 30, 2021. [↑](#footnote-ref-7)
8. James Politi, “Salvini Ramps up Rhetoric with Attack on Mandatory Vacccines.” *Financial Times*, June 22, 2018, <https://www.ft.com/content/e513740e-761a-11e8-b326-75a27d27ea5f>. [↑](#footnote-ref-8)
9. “Vaccini, Salvini Insiste: ‘Restiamo in Maggioranza, ma in Parlamento Veteremo Contro l’Obbligo.” *La Repubblica*, September 3, 2021. [↑](#footnote-ref-9)
10. “Vaccino Covid, Salvini: ‘No a Obbligo.” *Andkronos.com*, August 24, 2021, https://www.adnkronos.com/vaccino-covid-salvini-no-a-obbligo_2HLAP4rOItdLIXwEPQHiLf. [↑](#footnote-ref-10)
11. <https://www.facebook.com/legasalvinipremier/posts/3507696895939878/>. [↑](#footnote-ref-11)
12. Miles Johnson, “Italy Set to Make Covid ‘Green Pass’ Compulsory for All Workers.” *Financial Times*, September 16, 2021, <https://www.ft.com/content/4b7d00fa-2a3d-48a8-9a77-660474b5a423>. [↑](#footnote-ref-12)
13. Concetto Vecchio. “Scontro sul Green Pass, Salvini Tenta la Spallata: ‘Aboliamolo a Marzo.’” *La Repubblica*, February 14, 2022, <https://www.repubblica.it/cronaca/2022/02/14/news/scontro_sul_green_pass_salvini_tenta_la_spallata_aboliamolo_a_marzo_-337640456/>. [↑](#footnote-ref-13)
14. “Renato Brunetta: ‘Salvini Sbaglia, Io Farei Vaccino a Tutti Gli Statali.” *HuffPost*, July 25, 2021, <https://www.huffingtonpost.it/entry/renato-brunetta-salvini-sbaglia-io-farei-vaccino-a-tutti-gli-statali_it_60fd0230e4b0d2a22d4c233c/>. [↑](#footnote-ref-14)
15. “Super Green Pass piu Vicino, Regioni Compatte Contro I No Vax. Anticipo a 5 Mesi della Terza Dose di Vaccino. Giovedi’ il CDM.” *La Repubblica*, November 22, 2021, <https://www.repubblica.it/politica/2021/11/22/news/green_pass_regioni-327389389/>. [↑](#footnote-ref-15)
16. For example, in March 2022, former *M5S* Prime Minister Giuseppe Conte called for a scaling back of the super green pass, which would not allow the unvaccinated to work, while acknowledging that the decision of some citizens to remain unvaccinated at this juncture was “unjustifiable.” “Covid, Conte: ‘Stop al Super Green Pass nei Luoghi di Lavoro.’ Salvini: “Bene, la Lega non e’ piu Sola in Questa Battaglia.” *La Repubblica*, March 4, 2022. For a discussion of how *M5S* began to overcome its politically damaging early anti-vaccination stance before the pandemic, see: Carlo Martuscelli, “Italy’s 5 Star Movement Learns to Love Coronavirus Vaccines,” *Politico.eu*, January 4, 2021, <https://www.politico.eu/article/italy-5-star-movement-vaccine-coronavirus-beppe-grillo-anti-vax/>. [↑](#footnote-ref-16)
