# Supplementary material for: Resistance to COVID-19 vaccination and the social contract: evidence from Italy
Source: NPJ Vaccines. 2023 Apr 22;8:60. doi: 10.1038/s41541-023-00660-8 (PMC10122449; doi:10.1038/s41541-023-00660-8)
Supplement: Supplementary file 2 — REPORTING SUMMARY [file 41541_2023_660_MOESM2_ESM.pdf]

nature portfolio

Corresponding author(s): Dr Douglas (Lee) Kriner

Last updated by author(s): Mar 23, 2023

Reporting Summary

Nature Portfolio wishes to improve the reproducibility of the work that we publish. This form provides structure for consistency and transparency in reporting. For further information on Nature Portfolio policies, see our Editorial Policies and the Editorial Policy Checklist.

Statistics

For all statistical analyses, confirm that the following items are present in the figure legend, table legend, main text, or Methods section.

n/a	Confirmed	
☐	☒	The exact sample size (*n*) for each experimental group/condition, given as a discrete number and unit of measurement
☐	☒	A statement on whether measurements were taken from distinct samples or whether the same sample was measured repeatedly
☐	☒	The statistical test(s) used AND whether they are one- or two-sided<br>*Only common tests should be described solely by name; describe more complex techniques in the Methods section.*
☐	☒	A description of all covariates tested
☐	☒	A description of any assumptions or corrections, such as tests of normality and adjustment for multiple comparisons
☐	☒	A full description of the statistical parameters including central tendency (e.g. means) or other basic estimates (e.g. regression coefficient) AND variation (e.g. standard deviation) or associated estimates of uncertainty (e.g. confidence intervals)
☐	☒	For null hypothesis testing, the test statistic (e.g. *F*, *t*, *r*) with confidence intervals, effect sizes, degrees of freedom and *P* value noted<br>*Give P values as exact values whenever suitable.*
☒	☐	For Bayesian analysis, information on the choice of priors and Markov chain Monte Carlo settings
☒	☐	For hierarchical and complex designs, identification of the appropriate level for tests and full reporting of outcomes
☐	☒	Estimates of effect sizes (e.g. Cohen's *d*, Pearson's *r*), indicating how they were calculated

Our web collection on statistics for biologists contains articles on many of the points above.

Software and code

Policy information about availability of computer code

Data collection	Survey data was collected via the surey firm YouGov and analyzed using STATA 15.
Data analysis	All analyses were conducted using STATA 15. Complete code to replicate all tables and figures will be shared via the Harvard Dataverse upon publication.

For manuscripts utilizing custom algorithms or software that are central to the research but not yet described in published literature, software must be made available to editors and reviewers. We strongly encourage code deposition in a community repository (e.g. GitHub). See the Nature Portfolio guidelines for submitting code & software for further information.

Data

Policy information about availability of data

All manuscripts must include a data availability statement. This statement should provide the following information, where applicable:

- Accession codes, unique identifiers, or web links for publicly available datasets
- A description of any restrictions on data availability
- For clinical datasets or third party data, please ensure that the statement adheres to our policy

Upon publication, all data and code to replicate the analyses will be publicly available at the Harvard Dataverse: https://doi.org/10.7910/DVN/2V4IBP

Human research participants

Policy information about studies involving human research participants and Sex and Gender in Research.

Reporting on sex and gender	N/A.
Population characteristics	National sample of adult Italians.
Recruitment	Subjects were recruited by the survey firm YouGov.
Ethics oversight	Cornell University Institutional Review Board, protocol #2007009729

Note that full information on the approval of the study protocol must also be provided in the manuscript.

Field-specific reporting

Please select the one below that is the best fit for your research. If you are not sure, read the appropriate sections before making your selection.

☐ Life sciences ☒ Behavioural & social sciences ☐ Ecological, evolutionary & environmental sciences

For a reference copy of the document with all sections, see nature.com/documents/nr-reporting-summary-flat.pdf

Behavioural & social sciences study design

All studies must disclose on these points even when the disclosure is negative.

Study description	Analysis of original survey data in Italy and analysis of aggregate correlations between Italian regional vaccination rates and social capital scores generated by prior research.
Research sample	1,000 adult Italians, representative of the population as a whole.
Sampling strategy	YouGov usies stratificaiton and matching from their online panel. Additional details available here: https://today.yougov.com/about/panel-methodology/.
Data collection	Survey administered online by YouGov.
Timing	March 14-20, 2022
Data exclusions	None.
Non-participation	YouGov interviewed 1038 respondents who were then matched down to a sample of 1000 to produce the final dataset. The respondents were matched to a sampling frame on gender, age, and education. The frame was from 2019 Eurobarometer Survey.<br>The matched cases were weighted to the sampling frame using propensity scores. The matched cases and the frame were combined and a logistic regression was estimated for inclusion in the frame. The propensity score function included age, gender and education. The propensity scores were grouped into deciles of the estimated propensity score in the frame and post-stratified according to these deciles.<br>The weights were then post-stratified region, 2018 vote choice, and a three-way stratification of gender, age and education, to produce the final weight.
Randomization	Two questions in the survey were split-sample questions with subjects randomly assigned to one version or the other.

Reporting for specific materials, systems and methods

We require information from authors about some types of materials, experimental systems and methods used in many studies. Here, indicate whether each material, system or method listed is relevant to your study. If you are not sure if a list item applies to your research, read the appropriate section before selecting a response.

Materials & experimental systems

n/a	Involved in the study
☒	☐ Antibodies
☒	☐ Eukaryotic cell lines
☒	☐ Palaeontology and archaeology
☒	☐ Animals and other organisms
☒	☐ Clinical data
☒	☐ Dual use research of concern

Methods

n/a	Involved in the study
☒	☐ ChIP-seq
☒	☐ Flow cytometry
☒	☐ MRI-based neuroimaging
